# The Systemic Immune Signature of Cholesteatoma: A Comprehensive Cross-Sectional Analysis of Hematological Markers and Clinical Staging

**DOI:** 10.7759/cureus.112278

**Published:** 2026-07-08

**Authors:** Sahil Chauhan, Shaila Sidam, Vikas Gupta, Arati A Bhadade, Ujjawal Khurana, Anjan K Sahoo, Aparna Chavan, Ganakalyan Behera, Utkal P Mishra, Twisha Chatterjee

**Affiliations:** 1 Otolaryngology - Head and Neck Surgery, All India Institute of Medical Sciences, Bhopal, Bhopal, IND; 2 Otorhinolaryngology, All India Institute of Medical Sciences, Bhopal, Bhopal, IND; 3 Microbiology, All India Institute of Medical Sciences, Bhopal, Bhopal, IND; 4 Pathology and Laboratory Medicine, All India Institute of Medical Sciences, Bhopal, Bhopal, IND

**Keywords:** cholesteatoma, chronic otitis media, disease severity, erythrocyte sedimentation rate, inflammation, neutrophil-to-lymphocyte ratio, systemic immune-inflammation index

## Abstract

Background: Cholesteatoma is an inflammatory disease of the middle ear, which is characterized by hearing loss, bony destruction, and increased potential for complications. The inflammation plays a major role in the pathogenesis of disease; however, the relationship between systemic haematological inflammatory markers and disease severity remains inadequately understood. This study attempts to evaluate this association between conventional and novel inflammatory markers and the staging of cholesteatoma.

Methods: This study is a prospective cross-sectional analytical study, conducted at a tertiary care center in Central India over 18 months. Seventy-two patients with chronic otitis media (COM) squamosal disease undergoing tympanomastoid surgery and having confirmed or suspected cholesteatoma were included. The patients were further classified as per the European Academy of Otology and Neurotology/Japan Otological Society (EAONO/JOS 2017) staging system. The blood examinations done preoperatively were complete blood count, erythrocyte sedimentation rate (ESR), and C-reactive protein (CRP). The neutrophil-to-lymphocyte ratio (NLR) and Systemic Immune-Inflammation Index (SII) from complete blood count were calculated. Comparisons across the various disease stages were performed using the Kruskal-Wallis test, and correlations with disease stage were assessed using Spearman’s rank correlation.

Results: The age range was 6-55 years, with a mean age of 25.36 ± 11.56 years. There was no marked gender predominance observed in the study. As per staging, stage II disease was the most common presentation seen in 40 (55.6%) cases, followed by stage III in 26 (36.1%) cases. The median values of all inflammatory markers increased progressively with advancing stage of cholesteatoma. The Systemic Immune-Inflammation Index progressively increased from 286.5 (interquartile range (IQR): 276.75-424.00) in stage I to 1304.0 (IQR: 1163.0-1445.0) in stage IV. Although the neutrophil-to-lymphocyte ratio, erythrocyte sedimentation rate, and C-reactive protein showed upward trends with disease severity, interstage differences were not statistically significant. Spearman’s rank correlation coefficients (ρ) between inflammatory markers for the Systemic Immune-Inflammation Index and erythrocyte sedimentation ratio demonstrated statistically significant positive correlations with advancing disease stage.

Conclusion: The systemic inflammatory burden increases with advancing stage of cholesteatomatous otitis media. However, among the evaluated biomarkers, the Systemic Immune-Inflammation Index demonstrated the strongest and most significant association with disease severity. Hence, it has a potential utility as an inexpensive, readily available adjunctive marker for preoperative assessment and risk estimation in patients with cholesteatomatous chronic otitis media.

## Introduction

Cholesteatomatous otitis media is an important global health concern due to its chronic progressive nature, destructive potential, and association with morbidity. Although it is histologically benign, it behaves as a locally invasive disease capable of causing destruction to ossicles, diminished hearing, facial weakness, fistula in semicircular canals, meningitis, and brain abscess if not diagnosed and treated early [1,2]. The disease significantly affects quality of life worldwide, particularly in developing countries where access to specialized otologic care may be limited [3].

Chronic suppurative otitis media (CSOM), the cholesteatomatous form, affects approximately 65-330 million people worldwide. The prevalence is higher in low- and middle-income countries [3]. The estimated annual incidence is approximately 3 per 100,000 children and 9.2 cases per 100,000 adults [4]. Delayed presentation and recurrent infections result in advanced disease, which requires extensive surgical management and long-term follow-up.

In India, cholesteatoma constitutes a major otologic burden owing to the high prevalence of CSOM, poor socioeconomic conditions, overcrowding, malnutrition, recurrent upper respiratory tract infections, and inadequate healthcare access [5]. Several Indian studies have reported that patients commonly present at advanced stages of disease with extensive bone erosion and complications due to delayed healthcare-seeking behavior [6].

Hearing loss associated with cholesteatoma may adversely affect speech and language development in children, educational performance, and productivity in adults, thereby contributing to significant social and economic consequences [5,6].

Cholesteatoma is a destructive, keratinizing squamous epithelial lesion of the middle ear and mastoid, characterized by independent growth and progressive bone resorption [3]. The various agents responsible are cytokines, metalloproteinases, and receptor activator of nuclear factor kappa-B ligand (RANKL) [7].

The management of cholesteatoma often requires specialized otologic surgery and prolonged postoperative surveillance because of the risk of recurrence and residual disease [1]. Therefore, early detection and identification of markers reflecting disease severity are essential for improving clinical outcomes and reducing complications.

Acute inflammatory reactants such as erythrocyte sedimentation rate (ESR) and C-reactive protein (CRP) are widely used; however, their sensitivity in localized otologic diseases is limited. Hence, to measure systemic inflammatory status, the neutrophil-to-lymphocyte ratio (NLR) and Systemic Immune-Inflammation Index (SII) are emerging hematological markers [8]. These indices have shown potential utility in assessing disease severity, predicting complications, and correlating with clinical staging in chronic inflammatory diseases [9]. In recent years, attention has shifted toward derived hematological inflammatory indices obtained from routine complete blood count parameters, including NLR and SII. These markers have emerged as inexpensive, readily available, and potentially more sensitive indicators of systemic inflammatory status and disease severity [10].

Considering the paucity of data regarding the association between hematological inflammatory markers and cholesteatoma severity, the present study was undertaken to evaluate inflammatory hematological parameters in patients with cholesteatomatous otitis media and to determine their relationship with the clinical staging of cholesteatoma.

## Materials and methods

This was an institution-based cross-sectional analytical study. It was conducted in the Department of Otorhinolaryngology - Head and Neck Surgery of a tertiary care referral center in Central India over a period of 18 months. This study was reviewed and approved by the Institutional Human Ethics Committee of All India Institute of Medical Sciences (AIIMS), Bhopal, India (reference number: AIIMS/BPL/IHECSR/JAN/23/PG/19) before the commencement of the study. The study enrolled 72 patients diagnosed with chronic otitis media (COM) squamosal disease who either had visible cholesteatoma or were suspected of harboring cholesteatoma and subsequently underwent tympanomastoid surgery. Eligible participants included patients undergoing surgery for COM squamosal disease with either clinically evident cholesteatoma or a strong suspicion of cholesteatoma. Patients were excluded if they had received systemic or topical antibiotics or steroids within 15 days prior to surgery, had known systemic inflammatory or autoimmune disorders, refused consent, had undergone previous ear surgery, or were deemed unfit for surgery.

The severity and extent of cholesteatoma were assessed by the staging system described by the European Academy of Otology and Neurotology/Japan Otological Society (EAONO/JOS 2017) [11]. This classification categorizes cholesteatoma into four stages based on involvement of the attic (A), tympanic cavity (T), and mastoid (M), and the presence of complications (C). Stage I represents disease confined to the primary site of origin, whereas stage II indicates extension to two or more anatomical sites. Stage III includes cases associated with extracranial complications such as facial nerve palsy, labyrinthine fistula, or postauricular abscess, while stage IV comprises cases with intracranial complications, including meningitis, brain abscess, or lateral sinus thrombosis. Preoperative peripheral blood samples were collected 24-48 hours before to evaluate systemic inflammatory status. Laboratory parameters included erythrocyte sedimentation rate as a marker of chronic inflammation, acute phase response measured by C-reactive protein, total and differential leukocyte counts to determine absolute neutrophil and lymphocyte counts, and platelet counts to calculate composite inflammatory indices. The systemic inflammatory markers were calculated based on the complete blood count data. The SII was determined by multiplying the platelet and neutrophil counts and dividing the result by the lymphocyte count. The NLR was calculated by dividing the neutrophil count by the lymphocyte count.

Statistical analysis was performed using IBM SPSS Statistics for Windows version 29.0 (IBM Corp., Armonk, NY). Categorical variables were presented as numbers and percentages, while means ± standard deviations (SD) were calculated for continuous variables. Non-normally distributed variables were summarized as median and interquartile range (IQR). Differences in inflammatory markers, including SII [12], NLR [13], ESR, and CRP, across cholesteatoma stages were evaluated using the Kruskal-Wallis H test. For variables demonstrating statistically significant differences, pairwise post hoc comparisons with Bonferroni correction were conducted to identify the specific groups contributing to the observed differences. The association between inflammatory markers and cholesteatoma severity was examined using Spearman’s rank-order correlation analysis. A p-value of less than 0.05 was considered significant.

## Results

From the original cohort of 100 patients diagnosed with COM, based on the exclusion criteria, 28 participants were excluded. The excluded participants comprised three patients with a history of road traffic accidents resulting from head injury, 20 patients with various systemic comorbidities, 3 patients with a history of previous mastoid surgery, and 2 patients with usage of ear drops. These exclusions ensured a final study cohort of 72 patients while minimizing confounding factors that could impact the assessment of hematological inflammatory markers and their relation to the staging of cholesteatoma in cholesteatomatous otitis media.

Table 1 summarizes the demographic profile of the study participants. It included 72 participants with cholesteatomatous chronic otitis media in the final analysis. Their age ranged from 6 to 55 years, with a mean age of 25.36 ± 11.56 years. The maximum number of participants belonged to the 18-45 age group (52/72, 72.2%), followed by those aged less than 18 years (16/72, 22.2%). Only 4 patients (5.6%) were older than 45 years. The study population showed a nearly equal gender distribution, comprising 37 (51.4%) females and 35 (48.6%) males, with no marked gender predominance observed.

**Table 1:** Demographic Characteristics of the Study Participants (N = 72) SD: standard deviation

Characteristics	Category	Frequency (number)	Percentage (%)
Age group (years)	<18	16	22.2
	18-45	52	72.2
	>45	4	5.6
Age statistics	Mean ± SD	25.36 ± 11.56	-
	Range	6-55	-
Gender	Male	35	48.6
	Female	37	51.4

The distribution of cholesteatoma stages according to gender is presented in Table 2. Among the 72 study participants, stage II was the most common clinical stage, accounting for 40 (55.6%) patients, followed by stage III with 26 (36.1%) patients. Stage I and stage IV disease were less frequent, comprising 4 (5.6%) and 2 (2.8%) patients, respectively. Among patients with stage II disease, females constituted a slightly higher proportion than males (57.5% versus 42.5%). In contrast, stage III disease was more prevalent among males, who accounted for 61.5% of cases in this stage. Stage I disease showed an equal distribution between males and females. Notably, both patients with stage IV disease were female.

**Table 2:** Distribution of Clinical Stages According to Gender (N = 72)

Clinical stage	Male (number (%))	Female (number (%))	Total (number (%))
I	2 (50.0)	2 (50.0)	4 (5.6)
II	17 (42.5)	23 (57.5)	40 (55.6)
III	16 (61.5)	10 (38.5)	26 (36.1)
IV	0 (0.0)	2 (100.0)	2 (2.8)
Total	35 (48.6)	37 (51.4)	72 (100.0)

The median values of inflammatory markers according to cholesteatoma stage are presented in Table 3. Overall, SII was the only inflammatory marker that demonstrated a statistically significant association with cholesteatoma stage (H = 8.899, p = 0.031), which was performed using the Kruskal-Wallis test, whereas NLR, ESR, and CRP showed increasing trends that did not achieve statistical significance.

**Table 3:** Inflammatory Marker Levels According to Cholesteatoma Stage IQR: interquartile range, SII: Systemic Immune-Inflammation Index, NLR: neutrophil-to-lymphocyte ratio

Inflammatory marker	Stage I (median, IQR)	Stage II (median, IQR)	Stage III (median, IQR)	Stage IV (median, IQR)	H statistics	p-value
SII	286.5 (276.75-424.00)	472.0 (392.75-681.50)	666.5 (426.50-804.75)	1304.0 (1163.0-1445.0)	8.899	0.031
NLR	1.53 (1.15-1.97)	1.84 (1.28-2.42)	2.01 (1.45-2.93)	4.59 (3.33-5.85)	4.299	0.231
Erythrocyte sedimentation rate (mm/hr)	3.5 (2.0-6.25)	4.0 (2.0-7.25)	7.0 (4.0-8.75)	11.0 (6.5-15.5)	5.570	0.135
C-reactive protein (mg/L)	1.38 (1.17-2.08)	1.91 (0.48-3.25)	2.82 (0.90-6.94)	2.82 (0.90-6.94)	4.077	0.253

Table 4 depicts Spearman’s rank correlation coefficients (ρ) between inflammatory markers. A statistically significant positive correlation was observed between SII and cholesteatoma stage (ρ = 0.287, p = 0.015), indicating that higher SII values were associated with more advanced disease. Similarly, ESR demonstrated a significant positive correlation with cholesteatoma stage (ρ = 0.271, p = 0.021).

**Table 4:** Correlation Between Inflammatory Markers and Cholesteatoma Stage Correlation assessed using Spearman’s rank correlation coefficient Statistically significant at p < 0.05

Inflammatory marker	Spearman’s rho (ρ)	p-value	Interpretation
Systemic Immune-Inflammation Index	0.287	0.015	Significant
Neutrophil inflammation index	0.217	0.067	Non-significant
Erythrocyte sedimentation rate	0.271	0.021	Significant
C-reactive protein	0.200	0.093	Non-significant

## Discussion

Cholesteatomatous otitis media is a globally significant chronic otitis media because of its progressive inflammatory nature, potential for bone destruction, and risk for extracranial and intracranial complications. Identification of reliable biomarkers reflecting disease activity and severity may facilitate earlier risk stratification and improve clinical decision-making. In the present study, we evaluated conventional and novel hematological inflammatory markers and examined their association with cholesteatoma stage, with the aim of determining their utility as indicators of disease severity.

In the present study, the majority of cases occurred in the 18-45-year age group with a mean of 25.36 ± 11.56 years. This finding is consistent with previous studies reporting that acquired cholesteatoma predominantly affects adolescents and young adults. Recent epidemiological studies have similarly reported peak occurrence of cholesteatoma during the second and third decades of life, with mean ages ranging from approximately 21 to 31 years [14,15]. Pediatric cholesteatoma is generally more aggressive than adult disease, with faster growth, higher inflammatory activity, greater bone and ossicular erosion, poorer mastoid pneumatization, and increased rates of residual or recurrent disease. These differences are largely attributed to persistent Eustachian tube dysfunction and a more active inflammatory milieu in children. In this context, hematological inflammatory indices such as the neutrophil-to-lymphocyte ratio (NLR) and Systemic Immune-Inflammation Index (SII) may reflect the heightened systemic inflammatory burden seen in pediatric cases. Further age-stratified studies are needed to clarify whether these biomarkers can aid in distinguishing disease aggressiveness and guiding risk-based management in cholesteatoma [16].

Gender distribution in our cohort was nearly equal, with females accounting for 51.4% and males 48.6% of cases. Similar observations have been reported in studies demonstrating no significant gender predilection among patients with cholesteatoma [17]. While some epidemiological studies have reported a slight male predominance, the differences are generally modest and are unlikely to be of major clinical significance [15,17].

In the present study, stage II cholesteatoma was the most common presentation, accounting for more than half of all cases, followed by stage III disease. This finding suggests that most patients seek medical attention after the disease has progressed beyond the earliest stage, which is consistent with reports from developing countries where delayed healthcare access and prolonged duration of symptoms such as hearing impairment, persistent otorrhea, or complications often result in presentation at intermediate or advanced stages of cholesteatoma; in contrast, developed countries report a higher proportion of early-stage disease and fewer stage III cases due to earlier diagnosis and referral. This difference in case mix may have influenced our stage distribution and should be considered when interpreting the findings. However, it does not reduce the potential utility of hematological inflammatory markers, which may still serve as useful adjuncts for assessing disease burden across diverse healthcare settings [18-21].

Although female patients constituted a slightly higher proportion of stage II cases and both stage IV cases were female, stage III disease was more frequently observed among male patients. However, the overall gender distribution across stages did not demonstrate a consistent pattern, suggesting that disease severity may be influenced more by factors such as duration of disease, healthcare-seeking behavior, recurrent infections, and socioeconomic conditions rather than gender alone [15]. Similar studies have reported no significant association between gender and cholesteatoma stage or extent of disease at presentation [20,21].

The present study demonstrated a progressive increase in the median values of all evaluated inflammatory markers with advancing cholesteatoma stage. Among these markers, SII showed the most pronounced rise and was the only parameter significantly associated with disease stage. The median SII increased more than fourfold from stage I to stage IV disease, suggesting that systemic inflammatory burden increases in parallel with cholesteatoma severity. This finding may be biologically explained by the fact that cholesteatoma progression is driven by chronic inflammation characterized by infiltration of neutrophils, macrophages, and lymphocytes and the release of pro-inflammatory cytokines, which contributes to osteoclast activation and bone destruction [22-24].

SII integrates three hematological parameters (platelet count, neutrophil count, and lymphocyte count) and is considered a comprehensive marker of systemic inflammation. Hence, we interpret that the Systemic Immune-Inflammation Index (SII) may reflect disease activity more accurately than individual inflammatory markers, as it incorporates inflammatory, immune, and thrombotic responses. Elevated values of SII have been associated with chronic inflammatory conditions and more severe disease manifestations in otologic diseases. Therefore, these findings suggest that SII may warrant further investigation as a low-cost adjunct marker of disease severity in cholesteatoma, although prospective validation is needed before clinical application [25,26].

In the study, NLR demonstrated a progressive increase with advancing stage, but the difference did not reach statistical significance. Similar observations were also seen by many investigators who found elevated NLR values in patients with cholesteatoma compared with healthy controls, but inconsistent correlations with disease extent or severity [27,28].

The markers ESR and CRP also demonstrated increasing trends across disease stages; however, a statistically significant association was not seen with cholesteatoma severity. This finding might be due to the limitation of acute phase reactants in localized chronic inflammatory disorders. Similar findings were reported in previous studies of weak or inconsistent relationships between CRP, ESR, and cholesteatoma extent despite evidence of ongoing inflammatory activity within the lesion [24].

In the present study, all evaluated inflammatory markers tend to increase with advancing cholesteatoma stage; however, SII appears to be the most sensitive indicator of disease severity. It is cheap and widely available, with significant correlation with clinical staging, making it a useful adjunctive biomarker for assessment before surgery, along with risk prediction in patients with cholesteatomatous otitis media. However, to validate the findings, large multicentric studies are required to establish clinically relevant cutoff values for predicting advanced disease.

Spearman’s correlation analysis in the study demonstrated a significant positive association between cholesteatoma stage and both SII and ESR. Although NLR and CRP were also positive, they showed statistically non-significant correlations. Hence, increasing disease severity is associated with increasing systemic inflammatory response, and SII may be a more sensitive indicator of cholesteatoma progression than conventional inflammatory markers.

The association observed between SII and cholesteatoma stage may be possible due to the central role of chronic inflammation in cholesteatoma pathogenesis. Also, SII incorporates neutrophil, lymphocyte, and platelet counts, which reflect both inflammatory activation and immune status, providing a more comprehensive assessment of disease activity than individual hematological parameters alone [23].

Many studies have demonstrated elevated SII levels in patients with chronic otologic diseases. As per them, SII may be superior to traditional markers in reflecting disease severity and predicting adverse outcomes. The observed association in the present study further supports the potential role of SII as an inexpensive, readily available biomarker for preoperative assessment of cholesteatoma severity [25,26].

The inflammatory marker ESR also showed a significant positive correlation with disease stage, although the strength of association was slightly lower than SII. This finding supports the observation that systemic inflammatory activity increases with advancing cholesteatoma severity. However, ESR may be a nonspecific marker influenced by multiple physiological and pathological factors and therefore less reliable than composite inflammatory indices for assessing localized chronic inflammatory diseases [29].

The study showed that NLR and CRP demonstrated positive correlations with cholesteatoma stage, but the relationships did not achieve statistical significance. These findings have also been reported in previous studies, where NLR and CRP levels were elevated in patients with cholesteatoma compared with controls but showed inconsistent associations with disease extent or severity [27,28].

Overall, among the evaluated inflammatory markers, SII is the most useful hematological indicator of cholesteatoma severity. As it is simple, low cost, and available from routine complete blood counts, SII may be helpful to predict the risk and preoperative evaluation of patients with cholesteatomatous otitis media. To support this observation, a larger sample size is required to confirm the results and define clinically applicable cutoff values for identifying patients at risk of advanced disease.

The present study has several limitations. It was conducted at a single center with a modest sample size, which may limit generalizability. The cross-sectional design identifies associations but does not allow inference of causality between disease stage and systemic inflammatory markers. Although standardized operative and radiological criteria were used for staging, some observer variability cannot be excluded. In addition, despite excluding patients with known systemic illness, inflammatory indices such as NLR and SII may still have been influenced by subclinical or unrecognized conditions. Another limitation observed for NLR and CRP not reaching statistical significance might be due to small sample size, particularly in stage I and stage IV disease, or that localized middle-ear inflammation may not always produce sufficiently marked systemic changes to be detected by these markers. Also, there is a lack of follow-up data; hence, prospective longitudinal studies are required to determine whether these markers have prognostic value. These limitations highlight the need for larger, multicenter studies to validate and further refine these findings.

## Conclusions

In the present study, the systemic inflammatory markers tend to increase with advancing cholesteatoma stage, demonstrating the progressive inflammatory nature of cholesteatomatous otitis media. Among the analyzed biomarkers, the Systemic Immune-Inflammation Index showed a statistically significant association with disease stage and demonstrated the strongest correlation with cholesteatoma severity. Erythrocyte sedimentation rate also showed a significant positive correlation; however, NLR and CRP showed increasing trends but did not reach statistical significance.

These observations make SII a simple, inexpensive, and readily available biomarker for assessing disease severity in patients with cholesteatomatous otitis media. It can be derived from routine complete blood count parameters and may be useful as an adjunctive tool for preoperative evaluation, risk stratification, and identification of patients with more advanced disease. Hence, to establish our observations, large-scale, multicentric studies are required to clinically validate relevant cutoff values for the incorporation of SII into routine clinical practice.
